# Molecular Detection and Genetic Diversity of Cytomegaloviruses and Lymphocryptoviruses in Free-Roaming and Captive African Green Monkeys (*Chlorocebus sabaeus*)

**DOI:** 10.3390/ijms25063272

**Published:** 2024-03-14

**Authors:** Diana M. Mancuso, Kerry Gainor, Kerry M. Dore, Christa A. Gallagher, Amy Beierschmitt, Yashpal S. Malik, Souvik Ghosh

**Affiliations:** 1 Department of Biomedical Sciences, Ross University School of Veterinary Medicine, Basseterre P.O. Box 334, Saint Kitts and Nevis; dianamancuso@students.rossu.edu (D.M.M.); kerrygainor@students.rossu.edu (K.G.); kerrymdore@gmail.com (K.M.D.); cgallagher@rossvet.edu.kn (C.A.G.); abeierschmitt@rossvet.edu.kn (A.B.); 2 CABI/GEF/UNEP Regional Project—‘Preventing the COSTS of Invasive Alien Species in Barbados and OECS Countries’ in St. Kitts, Ministry of Environment, Climate Action and Constituency Development, Basseterre 00265, Saint Kitts and Nevis; 3 Science Department, Millbrook School, Millbrook, NY 12545, USA; 4 Behavioral Science Foundation, Estridge Estate, Basseterre P.O. Box 428, Saint Kitts and Nevis; 5 College of Animal Biotechnology, Guru Angad Dev Veterinary and Animal Science University, Ludhiana 141012, India; malikyps@gmail.com

**Keywords:** African green monkey (*Chlorocebus sabaeus*), cytomegalovirus, DNA polymerase, envelope glycoprotein B, herpesvirus, lymphocryptovirus

## Abstract

To date, limited information is available on cytomegalovirus (CMV) and lymphocryptovirus (LCV) from *Chlorocebus* monkeys. We report here high detection rates of herpesviruses in free-roaming African green monkeys (AGMs, *Chlorocebus sabaeus*) (26.4%, 23/87) and in captive AGMs (75%, 3/4) with respiratory disease on the Caribbean Island of St. Kitts. LCV (81.25%) was more prevalent than CMV (18.75%) in the AGMs. Applying a bigenic PCR approach (targeting DNA polymerase (DPOL) and glycoprotein B (gB) genes), long sequences were obtained from representative AGM CMV (KNA-SD6) and LCV (KNA-E4, -N6 and -R15) samples, and mixed LCV infections were identified in KNA-N6 and -R15. The nucleotide (nt) sequence (partial DPOL-intergenic region-partial gB) and partial DPOL- and gB-amino acid (aa) sequences of AGM CMV KNA-SD6 were closely related to *Cytomegalovirus cercopithecinebeta5* isolates from grivet monkeys, whilst those of AGM LCV KNA-E4 and -N6 (and E4-like gB of KNA-R15) were more closely related to cognate sequences of erythrocebus patas LCV1 from patas monkey than other LCVs, corroborating the concept of cospeciation in the evolution of CMV/LCV. On the other hand, the partial DPOL aa sequence of KNA-R15, and additional gB sequences (N6-gB-2 and R15-gB-2) from samples KNA-N6 and -R15 (respectively) appeared to be distinct from those of Old World monkey LCVs, indicating LCV evolutionary patterns that were not synchronous with those of host species. The present study is the first to report the molecular prevalence and genetic diversity of CMV/LCV from free-roaming/wild and captive AGMs, and is the first report on analysis of CMV nt/deduced aa sequences from AGMs and LCV gB sequences from *Chlorocebus* monkeys.

## 1. Introduction

Cytomegaloviruses (CMVs, genus *Cytomegalovirus*, subfamily *Betaherpesvirinae*) and lymphocryptoviruses (LCVs, genus *Lymphocryptovirus*, subfamily *Gammaherpesvirinae*) are double-stranded DNA viruses (genome size of ~196–241 kbp and ~149–171 kbp, respectively) within the family *Orthoherpesviridae* [1]. Based on virus isolation, serological and molecular studies, CMVs and LCVs have been reported in a wide variety of Old World monkeys (OWM) and New World monkeys (NWM) and are considered to be ubiquitous in non-human primate (NHP) populations [2,3,4,5,6,7,8,9,10,11]. However, many of these reports were from captive NHPs, whilst limited information is available on the molecular epidemiology of CMV and LCV in wild NHPs [2,3,4,5,6,7,8,9,10,11]. To date, 10 of the 11 recognized species within the genus *Cytomegalovirus* and 8 of the 9 recognized species within the genus *Lymphocryptovirus* are represented by simian herpesviruses [1]. In addition, partial sequences of several CMVs and LCVs from various NHPs have been designated as unclassified CMV/LCV viruses within the respective genera [1,12,13]. Although CMVs and LCVs have been shown to cause asymptomatic infections in immunocompetent NHPs, they have been associated with clinical conditions in immunosuppressed animals, such as CMV-associated necrotizing enteritis, encephalitis, lymphadenitis and/or pneumonitis and LCV-associated lymphomas and proliferative squamous epithelial lesions (resembling oral hairy leukoplakia in humans) in immunosuppressed macaques [4,5,6,7,8,9,10,14,15].

Cospeciations have been shown to play central roles during the evolution of CMVs and LCVs, as evident from phylogenetic analyses of partial, concatenated, and complete gene sequences, whilst CMV/LCV cross-species transmission/persistence are considered as rare events [2,3,11,16,17,18,19,20,21,22,23,24,25,26]. Phylogenetically, distinct monophyletic clades, separating OWM and NWM herpesviruses, have been observed for CMVs and LCVs, although the resolution of branching patterns were found to be incomplete within the OWM LCV clade, indicating a more complex evolutionary history of these viruses [2,3,11,21,24,25,26].

The green monkey/African green monkey (AGMs, genus *Chlorocebus sabaeus*, family *Cercopithecidae*) are members of the OWMs [27,28,29]. AGMs are native to Western parts of Africa and were introduced in the Caribbean islands during the 1600s [27,28]. Although members of the species *Chlorocebus aethiops* (related to *C. sabaeus*) have also been referred to as AGMs in previous publications on simian herpesviruses ([4,6,7,8,9,10] and several others), taxonomically, these NHPs are commonly known as grivet monkeys [29]. To date, limited information is available on the prevalence and genetic make-up/diversity of CMV and LCV in AGMs and other *Chlorocebus* monkeys [2,4,5,6,8,9,10,11,12,13,18,19,20,21].

Since the discovery of the first simian CMV in a kidney cell culture from a vervet monkey (*Chlorocebus pygerythrus*) in 1962 [30], only a handful of serological studies have investigated CMVs in *Chlorocebus* NHPs, revealing a high seroprevalence in grivet monkeys [31,32,33], whilst molecular epidemiological data are lacking. During industrial propagation of polioviruses, AGM kidney cell cultures were found to be frequently contaminated with simian CMVs, although the majority of the vaccine lots have been shown to be free of infectious CMVs [4,34,35]. The complete genome sequence (GenBank accession number FJ483968) of a single CMV isolate (isolate 2715, species *Cytomegalovirus cercopithecinebeta5* (CeBHV5)) has only been reported from *Chlorocebus* monkeys (grivet monkey) [1], whilst a few CMV DNA polymerase (DPOL) sequences are available from grivet monkeys (GenBank accession numbers AF292066, AY117754, JQ264771 and U63460) and from vervet monkeys (AY049065 and AY049066) [36].

A study in 1967 demonstrated cross-reactivity of AGM serum against human LCV (Epstein–Barr virus (EBV)) [37]. However, the isolation of the first LCV from *Chlorocebus* monkeys (from lymphoblasts of a grivet monkey) was reported in 1980 [38]. Since then, not much is known about LCVs in *Chlorocebus* monkeys [8,9,10,11], except for a few LCV DPOL sequences (222 bp) from illegally imported body parts (bone marrow and trachea) of AGMs [39] and the DPOL sequences (175 bp) of two unclassified LCV strains from grivet monkeys [19].

The Caribbean Island of St. Kitts (surface area of 176 square km) is inhabited by a large population of free-roaming AGMs (estimated to be ~30,000) that often come in contact with humans, companion animals, livestock, and other wildlife, providing an ideal environment for interspecies transmission of pathogens, as reported in previous studies [27,28,40,41,42,43,44,45,46,47,48]. The AGMs on St. Kitts destroy local agricultural production, impacting the island economy, and have been shown to negatively influence the island ecosystem [28,47,48]. As a result, these animals have been designated as alien invasive species, requiring efforts to control their growing population [27,47,48]. Considering the dearth of information on CMVs and LCVs in AGMs, the large population of AGMs on St. Kitts and their proximity to humans and other animals offered an excellent opportunity to study the prevalence, genetic diversity, and cross-species transmission of herpesviruses in free-roaming NHPs. In the present study, we report detection and molecular characterization of CMVs and LCVs in free-roaming and captive AGMs on St. Kitts. To our knowledge, this is the first report on molecular prevalence and genetic diversity of CMVs and LCVs in free roaming/wild and captive AGMs.

## 2. Results

### 2.1. Detection of CMVs and LCVs in AGMs

**Figure 1:**
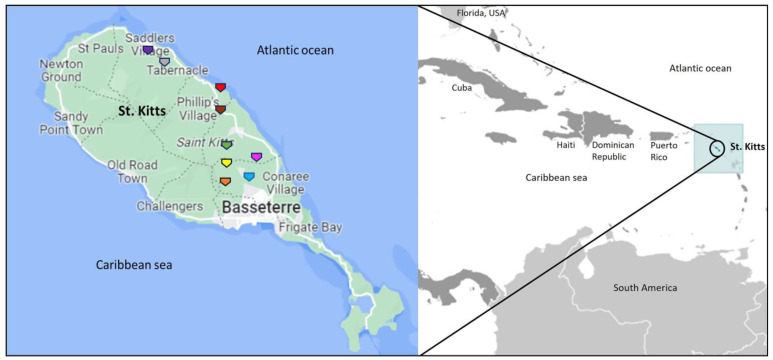
Map showing geographical location of the Island of St. Kitts in the Caribbean region. The map was adapted from https://www.cia.gov/the-world-factbook/countries/saint-kitts-and-nevis/locator-map (accessed on 15 May 2023) (**Right**). Map of St. Kitts showing the locations of the green monkey trapping sites (Bourreaux village, Cedar grove, Dale Mountain, Fountain Mountain, Green hill, Harris Mountain Forest, Monkey hill, and Saddlers is shown with a brown, orange, pink, yellow, green, grey, blue, and purple pentagon arrow, respectively) and that of the Behavioral Science Foundation (shown with a red pentagon arrow). The map of St. Kitts was adapted from https://www.google.com/maps (accessed on 15 May 2023) (**Left**).

The present study was based on AGM samples collected for a research project on simian adenoviruses (AdVs) [40]. The AGM trapping locations on St. Kitts are shown in Figure 1. A total of 23 (26.4%) of the 87 free-roaming AGMs tested positive for herpesviruses using a pan-herpesvirus DPOL nested PCR assay [49]. Herpesvirus DNA was detected in 16 nasal swabs and 8 rectal swabs, of which a single nasal swab and a single rectal swab were from the same AGM (KNA-44) (Table 1). The rates of detection of herpesviruses in juvenile and adult AGMs was 33% (5/15) and 25% (18/72), respectively, and 28.3% (19/67) and 20% (4/20) in AGMs trapped in/near human habitats and in the wild, respectively (Table 1). Among the archival samples, three (1 nasal swab each from two AGMs, and a nasal swab and an oral sample from a single AGM) of the four captive AGMs with severe respiratory disease tested PCR positive for herpesviruses, of which two animals had died (Table 1). Taken together, a total of 28 samples (19 nasal swabs, 8 rectal swabs and a single oral sample) from 26 AGMs were positive for herpesviruses (Table 1). Among the herpesvirus-positive AGMs, four of the free-roaming NHPs tested positive for AdVs, whilst none of the archival samples from the captive NHPs were PCR positive for AdVs.

**Figure 2:**
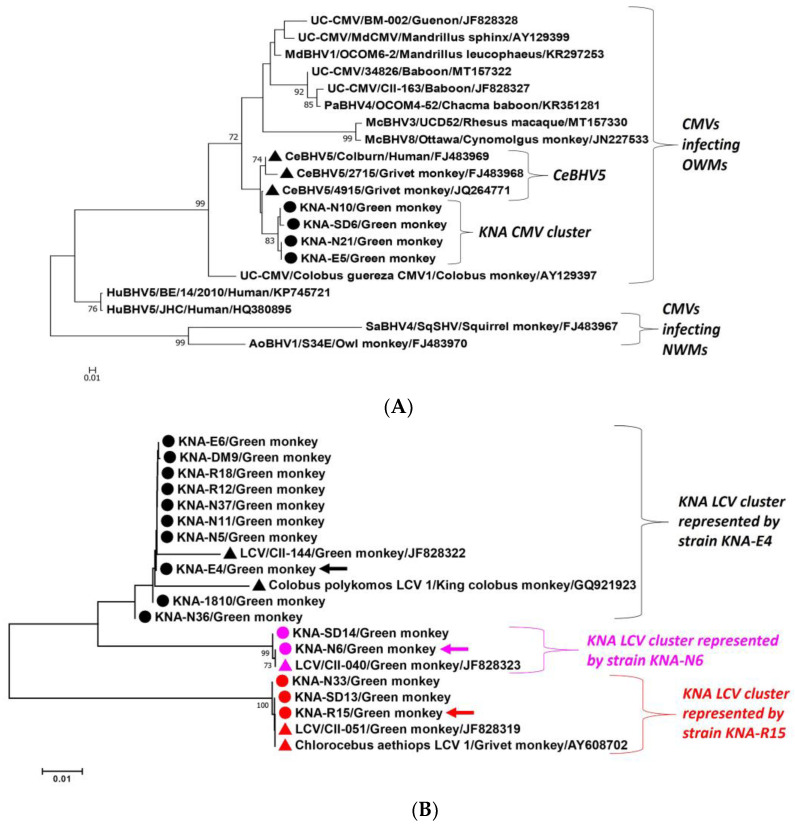
(**A**,**B**). Phylogenetic analysis of the partial DPOL coding sequences (~130 bp) of herpesviruses from green monkeys on St. Kitts (shown with circles) with cognate sequences of cytomegaloviruses (**A**) and lymphocryptoviruses (**B**) from primates. (**A**): Herpesvirus DPOL sequences obtained from samples KNA-E5, -N10, -N21 and -SD6 are highlighted with black circles, whilst CeBHV5 isolates/strains are shown with black triangles. (**B**): Herpesvirus DPOL sequences belonging to the KNA-E4-like, KNA-N6-like, and KNA-R15-like LCV cluster are shown with black, pink, and red circles/triangles, respectively. (**A**,**B**): The name of the virus/host of detection/GenBank accession number have been mentioned for the CMV/LCV sequences retrieved from the GenBank database. Bootstrap values < 70% are not shown. Scale bar, 0.01 substitutions per nucleotide. Abbreviations: AoBHV, Aotine betaherpesvirus; CeBHV, Cercopithecine betaherpesvirus; CMV, cytomegalovirus; HuBHV, Human betaherpesvirus; McBHV, Macacine betaherpesvirus; MdBHV, Mandrilline betaherpesvirus; NWMs, New World Monkeys; OWMs, Old World Monkeys; PaBHV, Papiine betaherpesvirus; LCV, lymphocryptovirus; SaBHV, Saimiriine betaherpesvirus; UC-CMV, Unclassified cytomegalovirus.

The screening PCR amplicons were sequenced to determine the nature (subfamily/genus) of the AGM herpesviruses. By BLASTN analysis, the partial DPOL sequences (~130 nucleotide (nt) residues) from 16 and 4 AGM samples shared maximum homology with LCV and CMV, respectively, whilst the remaining 8 PCR-positive samples were excluded from further analysis, as the respective DPOL sequences lacked quality (Phred value < 40) (Table 1). The four CMV-like DPOL sequences (KNA-E5, KNA-N10, KNA-N21 and KNA-SD6) shared 100% nt sequence identities between themselves. With other CMVs, the representative AGM CMV sequence, KNA-SD6, shared maximum nt identity of 96.97% with cognate sequence of CeBHV5 strain 4915 (Genbank accession number JQ264771) from a grivet monkey. Phylogenetically, the partial DPOL sequences of KNA-E5, -N10, -N21 and -SD6 grouped together within a cluster that consisted of CeBHV5 strains/isolates (Figure 2A). On the other hand, based on nt sequence identities and phylogenetic analysis, the LCV-like partial DPOL sequences from the AGMs formed three groups, represented by KNA-E4, KNA-N6 and KNA-R15, respectively (Figure 2B). Absolute nt sequence identities were observed between DPOL sequences within an LCV group, whilst identity of 94.57%, 90.70%, and 89.15% was observed between the representative AGM LCV sequences (KNA-E4 vs. KNA-N6, KNA-E4 vs. KNA-R15, and KNA-N6 vs. KNA-R15, respectively). With the other LCVs, KNA-E4, -N6 and -R15 shared maximum nt sequence identity of 98.48%, 100%, and 100% with the partial DPOL sequences of LCVs (strains CII-144, CII-040, and CII-051, respectively) from smuggled body parts of AGMs [39]. Interestingly, the nasal swab and oral discharge from AGM KNA-44 tested positive for CMV and LCV, respectively (Table 1).

Previous studies on evolution of simian CMVs and LCVs have relied on a bigenic PCR approach based on amplification of the conserved DPOL and envelope glycoprotein B (gB) genes [3,11,16,19,20,21,25]. Since the partial DPOL sequences (~130 nt) were not sufficient to gain a proper understanding of the genetic make-up/diversity of the AGM herpesviruses, longer DPOL and gB coding sequences (CDS) were obtained from the representative AGM CMV (KNA-SD6) and LCV (KNA-E4, KNA-N6 and KNA-R15) samples.

### 2.2. Analysis of the CMV DPOL and gB Coding Sequences from AGM Sample KNA-SD6

**Figure 3:**
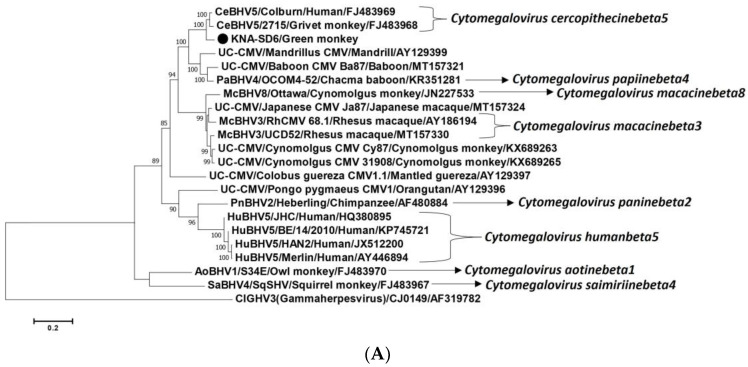

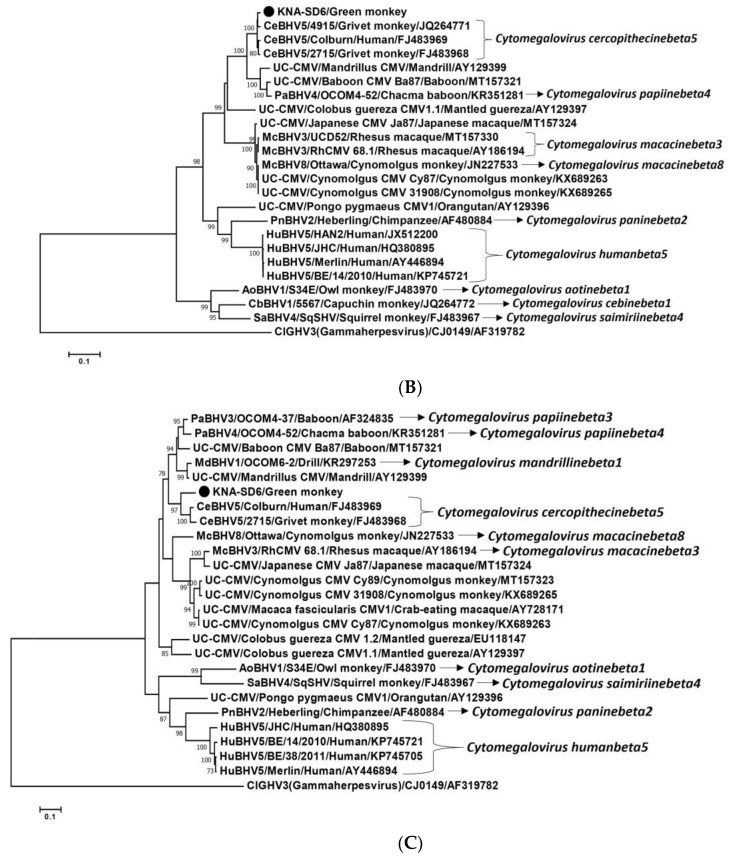
(**A**–**C**). Phylogenetic analysis of the partial nucleotide (nt) sequence (3688 nt, containing partial DPOL CDS-intergenic region-partial gB CDS) (**A**), partial deduced amino acid (aa) sequence of putative DPOL (691 aa) (**B**), and partial deduced aa sequence of putative gB (531 aa) (**C**) of herpesvirus strain KNA-SD6/Green monkey (shown with a black circle) with cognate sequences of CMVs from humans and non-human primates. (**A**–**C**): The recognized species within the genus *Cytomegalovirus* are shown with *italic* font. The name of the virus/name of the strain, or isolate/host of detection/GenBank accession number have been mentioned for the CMV sequences retrieved from the GenBank database. A member of the genus *Lymphocryptovirus* (ClGHV3/CJ0149/AF319782) was used as the outgroup sequence. Bootstrap values < 70% are not shown. Scale bar, 0.2 substitutions per nt (**A**), and 0.1 substitutions per aa residue (**B**,**C**). Abbreviations: AoBHV, Aotine betaherpesvirus; CDS, coding sequence; CeBHV, Cercopithecine betaherpesvirus; ClGHV, Callitrichine gammaherpesvirus; CMV, cytomegalovirus; DPOL, DNA polymerase; gB, envelope glycoprotein B; HuBHV, Human betaherpesvirus; McBHV, Macacine betaherpesvirus; MdBHV, Mandrilline betaherpesvirus; PaBHV, Papiine betaherpesvirus; PnBHV, Panine betaherpesvirus; SaBHV, Saimiriine betaherpesvirus; UC-CMV, Unclassified cytomegalovirus.

In the present study, we obtained a single CMV sequence (3688 nt, corresponding to nt 74797–nt 78484 of CeBHV5 isolate 2715, containing partial DPOL CDS-intergenic region-partial gB CDS) from AGM sample KNA-SD6 using primers described in Supplementary Table S1. On the other hand, CMV-positive AGM samples KNA-E5, -N10 and -N21 (shared partial DPOL (~130 bp) nt sequence identities of 100% with KNA-SD6) were not available in sufficient volumes, and therefore, could not be subjected to further molecular characterization. The KNA-SD6 sequence shared nt sequence identities of 93.10% and 93.15% with cognate sequences of CeBHV5 isolate 2715 and Colburn (GenBank accession number FJ483969, a CeBHV5 from brain biopsy sample of a boy with encephalopathy), respectively, followed by <75% identities with other herpesviruses. The partial DPOL (691 aa) of KNA-SD6 shared deduced aa identities of 97.83–98.55% with cognate sequences of CeBHV5 strain/isolates (isolate 2715 and strain 4915 from grivet monkeys and isolate Colburn), followed by identities of <90% with other CMVs. On the other hand, the partial gB (531 aa) of KNA-SD6 shared deduced aa identities of 87.80–88.37% with cognate sequences of CeBHV5 isolate 2715 and Colburn, and identities of <81% with other CMV sequences. By phylogenetic analysis, the partial nt and deduced aa sequences (DPOL and gB) of KNA-SD6 clustered with those of CeBHV5 isolates/strains (Figure 3A–C).

### 2.3. Analysis of the LCV DPOL and gB Coding Sequences from AGM Samples KNA-E4, -N6 and -R15

**Figure 4:**
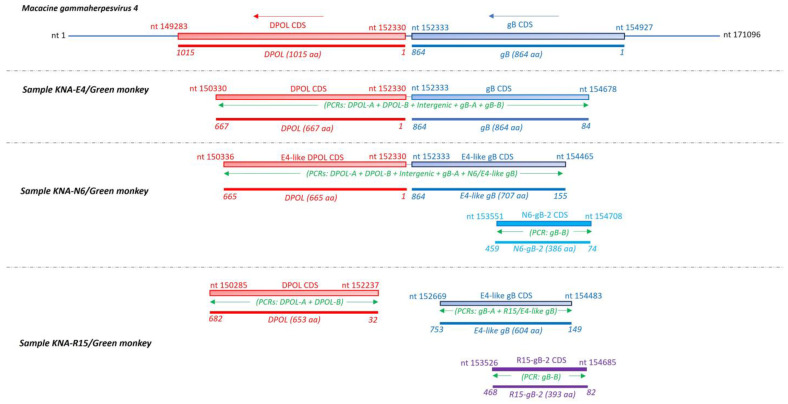
Gammaherpesvirus sequences obtained from green monkeys (GM) on St. Kitts. The PCR assays (Supplementary Table S1) employed to obtain the herpesvirus sequences are shown with green font. The nucleotide (nt)/amino acid (aa) positions shown for a GM herpesvirus sequence are those at corresponding positions of the nt/aa sequence of macacine gammaherpesvirus 4 (GenBank accession number AY037858). The direction (5-′ to 3-′, reverse strand) of the putative envelope glycoprotein B (gB) and DNA polymerase (DPOL) coding sequence (CDS) of macacine gammaherpesvirus 4 is shown with a blue and a red arrow, respectively.

Considering the complexity of the LCV sequence data obtained in the present study, a schematic representation of the AGM LCV sequences determined from samples KNA-E4, -N6 and -R15 is shown in Figure 4. The PCR assays/strategies employed to obtain the AGM LCV sequences were designed in the present study, and are shown in Figure 4 and described in Supplementary Table S1. A single LCV sequence (4340 nt, containing partial DPOL CDS-intergenic region-partial gB CDS) was obtained from sample KNA-E4 (Figure 4). On the other hand, two different LCV sequences: **(i)** a E4-like N6 sequence (4121 nt, consisting of partial DPOL CDS-intergenic region-partial gB CDS, sharing 97.62% identity with KNA-E4), and **(ii)** a partial gB CDS, designated as N6-gB-2 (1158 nt, 82.57% nt sequence identity with cognate stretch of E4-like N6 sequence), were derived from sample KNA-N6 (Figure 4). A single DPOL CDS (1959 nt, 86.23% identity with cognate sequence of KNA-E4), and two different gB CDS: **(i)** a E4-like R15 gB CDS (1812 nt, 99.28% identity with cognate sequence of KNA-E4), and **(ii)** a second gB CDS, designated as R15-gB-2 (1182 nt, 78.79% identity with cognate stretch of E4-like R15 gB CDS), were obtained from sample KNA-R15 (Figure 4). Sample KNA-R15 did not exhibit amplification with nested PCRs using R15 DPOL-specific forward primers and E4-like R15 gB-, or R15-gB-2-specific reverse primers. Furthermore, although a E4-like gB CDS was identified in KNA-R15, nested-PCRs with E4-specific DPOL primers (as well as the 1st round PCR, and PCRs using combination of forward and reverse primers from the 1st and 2nd round PCRs, respectively, and vice versa) yielded negative results, which might be due to accumulation of mutations at the primer binding sites, or possible recombination events. Taken together, these observations suggested that at least two different LCV strains might be present in sample KNA-N6 and KNA-R15, respectively.

**Figure 5:**
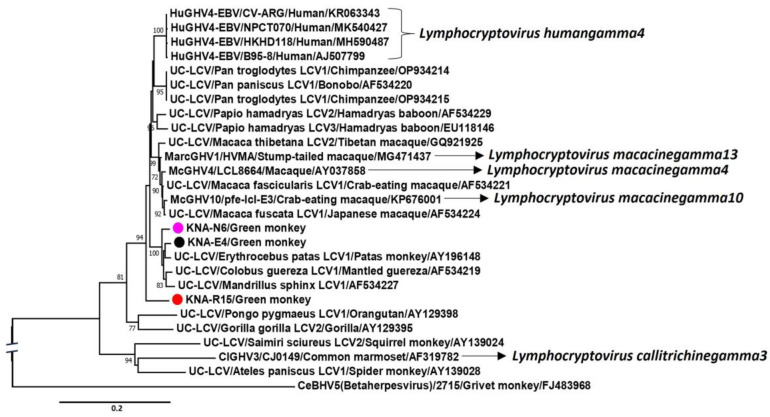
Phylogenetic analysis of the partial deduced amino acid (aa) sequences (~635 aa) of the putative DPOL of herpesviruses from green monkeys (samples KNA-E4, -N6 and -R15 is shown with black, pink, and red circle, respectively) on St. Kitts with cognate sequences of LCVs from humans and non-human primates. To rule out biases, cognate DPOL sequences of KNA-E4 (partial DPOL sequence was 667 aa in size; however, aa 32–aa 665 was included in the analysis), -N6 (665 aa; aa 32–aa 665), and -R15 (653 aa; aa 1–aa 636) were included in the analysis. The recognized species within the genus *Lymphocryptovirus* are shown with *italic* font. The name of the virus/name of the strain, or isolate/host of detection/GenBank accession number have been mentioned for the LCV sequences retrieved from the GenBank database. A member of the genus *Cytomegalovirus* (CeBHV5/2715/Grivet monkey/FJ483968) was used as the outgroup sequence. Bootstrap values < 70% are not shown. Scale bar, 0.2 substitutions per aa residue. Abbreviations: CeBHV, Cercopithecine betaherpesvirus; ClGHV, Callitrichine gammaherpesvirus; DPOL, DNA polymerase; HuGHV, Human gammaherpesvirus; LCV, lymphocryptovirus; MarcGHV, Macaca arctoides gammaherpesvirus; McGHV, Macacine gammaherpesvirus; UC-LCV, Unclassified lymphocryptovirus.

The partial deduced aa sequence (667 aa) of the putative DPOL of KNA-E4 shared identity of 97.74% and 90.68% with cognate stretch of partial DPOL sequences of KNA-N6 (665 aa) and -R15 (653 aa), respectively. With other LCVs, the DPOL aa sequences of KNA-E4 and -N6 shared identities of 96.54–97.75% with cognate sequences of unclassified LCVs from NHPs: erythrocebus patas LCV1 (GenBank accession number AY196148), colobus guereza LCV1 (AF534219) and mandrillus sphinx LCV1 (AF534227), followed by identities of ~94% with several human gammaherpesvirus4 (HuGHV4) strains/isolates. On the other hand, the DPOL of KNA-R15 shared a maximum aa identity of 93.27% with cognate sequence of macaca arctoides gammaherpesvirus1 (MarcGHV1) isolate HVMA (MG471437), followed by identities of ~92% with macacine gammaherpesvirus4 (McGHV4) strain LCL8664 (AY037858) and several HuGHV4 strains/isolates. Phylogenetically, the partial DPOL aa sequences of KNA-E4, -N6, erythrocebus patas LCV1, colobus guereza LCV1 and mandrillus sphinx LCV1 formed a single group, within which KNA-E4 and erythrocebus patas LCV1 clustered together, whilst the DPOL of KNA-R15 formed a distinct branch within the clade of OWM LCVs (Figure 5). A hypothetical time tree estimating the divergence times of KNA-E4, -N6 and –R15 and those of LCVs from other primates has been shown in Supplementary Figure S1. Although a few LCV DPOL sequences have been previously reported from Chlorocebus monkeys including AGMs, they were only 175 bp [19] or 222 bp [39] in size, and therefore, excluded from phylogenetic analysis.

Since the partial deduced aa sequence of the putative gB of KNA-E4 shared > 99% identities with the E4-like gB sequences from samples KNA-N6 and -R15, and the E4 gB sequence (778 aa) was longer in size compared to the E4-like-N6 (707 aa) and E4-like-R15 (604 aa) gB sequences, only the gB from sample KNA-E4 was included for determining deduced aa identities. By BLASTP analysis, the gB of KNA-E4 shared a maximum deduced aa identity of 94.47% with cognate stretch of the gB sequence of papiine gammaherpesvirus1 (PaGHV1, AJ581750) from baboon lymphoblastoid cell line 594S, followed by <90% identities with simian and human LCVs. However, a deduced aa identity of 97.78% was observed between corresponding regions (aa 464–aa 778 and aa 1–aa 315, respectively) of the partial gB sequence of KNA-E4 and the partial gB sequence (315 aa in size) of erythrocebus patas LCV1.

**Figure 6:**
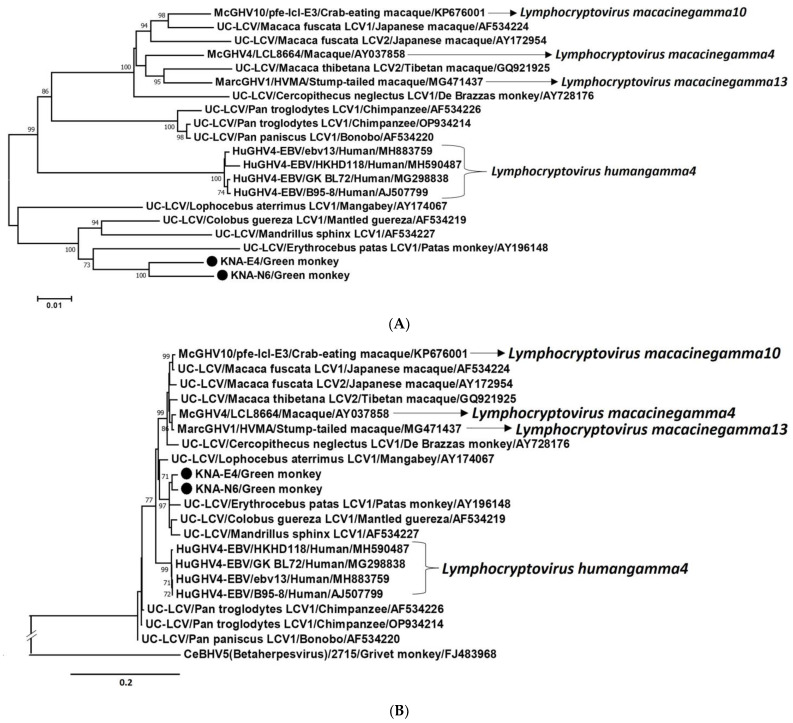
(**A**,**B**). Phylogenetic analysis of the partial nucleotide (nt) sequences (partial DPOL CDS-intergenic region-partial gB CDS) (**A**) and concatenated deduced amino acid (aa) sequences (partial gB-partial DPOL) (**B**) of gammaherpesviruses from samples KNA-E4/Green monkey and KNA-N6/Green monkey (shown with black circles) with cognate sequences of LCV strain erythrocebus patas LCV1 and other LCVs from humans and non-human primates. Since variable lengths of nt/deduced aa sequences were available for the LCV strains, to rule out biases during phylogenetic analysis, the KNA-E4, E4-like KNA-N6, erythrocebus patas LCV1, and other LCV sequences were trimmed following multiple alignment. The trimmed nt sequences were ~2945 nt in size, whilst the trimmed gB and DPOL sequences were ~315 aa and ~665 aa in size, respectively. Scale bar, 0.01 substitutions per nt residue (**A**), and 0.2 substitutions per aa residue (**B**). (**A**,**B**): The recognized species within the genus *Lymphocryptovirus* are shown with *italic* font. The name of the virus/name of the strain, or isolate/host of detection/GenBank accession number have been mentioned for the LCV sequences retrieved from the GenBank database. Bootstrap values < 70% are not shown. (**B**): A member of the genus *Cytomegalovirus* (CeBHV5/2715/Grivet monkey/ FJ483968) was used as the outgroup sequence. Abbreviations: CDS, coding sequence; DPOL, DNA polymerase; gB, envelope glycoprotein B; HuGHV, Human gammaherpesvirus; LCV, lymphocryptovirus; MarcGHV, Macaca arctoides gammaherpesvirus; McGHV, Macacine gammaherpesvirus; UC-LCV, Unclassified lymphocryptovirus.

To better understand the genetic relationship between KNA-E4, E4-like KNA-N6 and erythrocebus patas LCV1, cognate nt sequences (partial DPOL CDS-intergenic region-partial gB CDS) as well as concatenated deduced aa sequences (gB-DPOL) from these LCVs were subjected to phylogenetic analysis. Since variable lengths of LCV sequences were available in the GenBank database (https://www.ncbi.nlm.nih.gov/nuccore/, accessed 20 December 2023), to rule out biases during phylogenetic analysis, we trimmed the KNA-E4, E4-like KNA-N6, erythrocebus patas LCV1 and other LCV nt/aa sequences following multiple alignment. Phylogenetically, the nt and concatenated deduced aa sequences of KNA-E4 and -N6 clustered near erythrocebus patas LCV1 within a cluster that also consisted of colobus guereza LCV1 and mandrillus sphinx LCV1 (Figure 6A,B), corroborating the observations based on deduced aa identities. Furthermore, nt sequence identities of ~94% were observed between the overlapping regions (overlap of ~2945 bp) of KNA-E4/-N6 and erythrocebus patas LCV1 sequences, whilst by unbiased BLASTN analysis, the obtained KNA-E4 (4340 nt) and -N6 (4121 nt) sequences shared a maximum, yet low identity of 88.26%, respectively, with cognate sequence of McGHV4 strain LCL8664. We did not find any evidence for recombination events involving the KNA-E4 and -N6 nt sequences.

**Figure 7:**
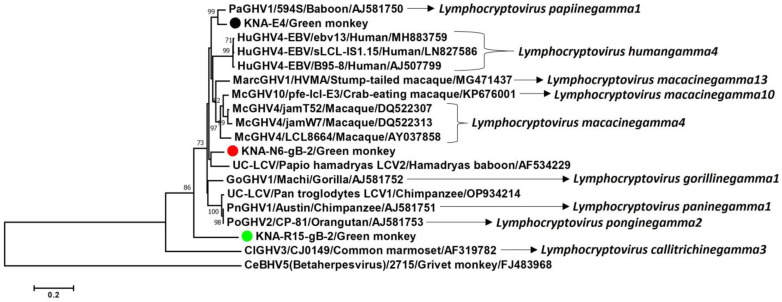
Phylogenetic analysis of the KNA-E4, KNA-N6-gB-2 and KNA-R15-gB-2 gB sequences (shown with black, red, and green circles, respectively) with cognate deduced amino acid (aa) sequences of LCVs from humans and non-human primates. To rule out biases, the partial gB sequences of KNA-E4 (778aa in size; however, aa 1–aa 375 was included in the analysis), KNA-N6-gB-2 (386 aa; aa 11–aa 386) and KNA-R15-gB-2 (393 aa; aa 3–aa 381) were trimmed following multiple alignment, and phylogenetic analysis was based on ~375 aa gB sequences. Although the partial nucleotide and concatenated deduced aa sequences (partial gB-partial DPOL) of KNA-E4 were closely related to those of erythrocebus patas LCV1 (Figure 6A,B), the partial gB aa sequence (aa 1–aa 315) of erythrocebus patas LCV1 overlapped with aa 464–aa 778 of putative gB of KNA-E4. As a result, the gB aa sequence of erythrocebus patas LCV1 was not included in the analysis, and in Figure 7, KNA-E4 clustered near an LCV from a baboon. The recognized species within the genus *Lymphocryptovirus* are shown with italic font. The name of the virus/name of the strain, or isolate/host of detection/GenBank accession number have been mentioned for the LCV sequences retrieved from the GenBank database. A member of the genus *Cytomegalovirus* (CeBHV5/2715/Grivet monkey/FJ483968) was used as the outgroup sequence. Bootstrap values < 70% are not shown. Scale bar, 0.2 substitutions per aa residue. Abbreviations: CeBHV, Cercopithecine betaherpesvirus; ClGHV, Callitrichine gammaherpesvirus; gB, envelope glycoprotein B; GoGHV, Gorilline gammaherpesvirus; HuGHV, Human gammaherpesvirus; LCV, lymphocryptovirus; MarcGHV, Macaca arctoides gammaherpesvirus; McGHV, Macacine gammaherpesvirus; PaGHV, Papiine gammaherpesvirus; PnGHV, Panine gammaherpesvirus; PoGHV, Pongine gammaherpesvirus; UC-LCV, Unclassified lymphocryptovirus.

The partial N6-gB-2 (386 aa) and R15-gB-2 (393 aa) gB sequences (additional gB sequence from sample KNA-N6 and KNA-R15, respectively) shared a deduced aa identity of 70.89% between themselves, and identities of 84.24–86.17% and 68.10–71.61%, respectively, with the E4-like-gB sequences (KNA-E4, E4-like-N6 and E4-like-R15). By BLASTP analysis, N6-gB-2 and R15-gB-2 shared a maximum deduced aa identity of 87.31% and 73.60% with cognate gB sequence of *Lymphocryptovirus macacinegamma10*/macacine gammaherpesvirus10 (McGHV10) isolate pfe-lcl-E3 (KP676001) from a crab-eating macaque and *Lymphocryptovirus ponginegamma2*/pongine gammaherpesvirus2 (PoGHV2, AJ581753) from orangutan lymphoblastoid cell line CP-81, respectively. Phylogenetically, N6-gB-2 clustered near unclassified LCV strain papio hamadryas LCV2, although the cluster was characterized by a low bootstrap value, whilst R15-gB-2 formed a distinct branch outside the OWM LCV clade (Figure 7).

## 3. Discussion

Since the first report on isolation of CMV and LCV from a vervet monkey and a grivet monkey in 1962 and 1980, respectively, little information is available about these viruses in Chlorocebus NHPs, especially from AGMs (*Chlorocebus sabeous*) [2,4,5,6,8,9,10,11,12,13,18,19,20,21,30,38]. To our knowledge, the present study is the first to report molecular prevalence and genetic diversity of CMV and LCV in free-roaming/wild and captive AGMs. Overall, we reported high rates of detection (26.4%, 23/87) of herpesviruses in the free-roaming AGMs on St. Kitts, corroborating previous observations that herpesviruses are widely distributed in OWMs [2,4,5,6,8,9,10,11,12,13,18,19,20,21]. Based on the partial DPOL sequences (n = 16) available for BLASTN analysis, the LCV and CMV detection rates were 81.25% (13/16) and 18.75% (3/16), respectively, indicating that LCVs might be more prevalent than CMVs in the St. Kitts AGM population. Although the samples were screened by a broad-spectrum PCR assay that has been used to amplify the DPOL of alpha-, beta- and gammaherpesviruses [49], we did not detect any members of the subfamily *Alphaherpesvirinae* in the free-roaming AGMs. Since eight of the partial DPOL sequences lacked quality (phred value < 40) and could not be included for homology search for cognate herpesvirus sequences, we could not entirely rule out higher CMV detection rates, or presence of alphaherpesviruses in these AGM samples. All the free-roaming AGMs that tested PCR positive for herpesviruses appeared to be apparently healthy, mirroring previous findings that herpesviruses usually cause asymptomatic infections in immunocompetent NHPs [4,5,6,7,8,9,10,14,15,50].

Interestingly, archival samples from three of the four captive AGMs with clinical signs of peracute respiratory disease tested positive for herpesviruses (LCV in nasal swabs from 2 AGMs that eventually died, and mixed infection with LCV (nasal swab) and CMV (oral discharge) in a single AGM) (Table 1). Necropsy of the dead NHPs revealed severe interstitial pneumonia (photographs were not available). Since samples were not available from the dead NHPs, we could not screen the lungs/other organs for presence of herpesviruses. Although the pathogenesis of CMV and LCV in AGMs remain to be properly elucidated [4,5,9], dissemination of CMVs have been associated with pneumonitis in immunosuppressed macaques and humans [4,5,51], whilst lymphocytic interstitial pneumonia has been reported in LCV-positive mountain gorilla infants [52] and in EBV infections in immunosuppressed and rarely in immunocompetent humans [53,54,55]. It might be possible that the LCV/CMV-positive captive AGMs were immunosuppressed due to infections with other pathogens, such as simian immunodeficiency virus (SIV), and/or from other stressful conditions (changing social dynamics, separation, and/or transportation), which might have resulted in clinical respiratory illness. Alternatively, the severe respiratory disease might have been caused by common respiratory pathogen/s, which subjected the captive AGMs to stress, resulting in shedding of herpesviruses. Although the four captive AGMs tested negative for AdVs, sufficient volumes of samples were not available for screening other respiratory pathogens (and SIV), and therefore, we could not establish possible roles of LCV/LCV + CMV in respiratory disease in these NHPs.

To date, a lack of sufficient sequence data has hindered studies on the genetic diversity and evolution of CMV and LCV from Chlorocebus NHPs, especially in AGMs (only four LCV partial DPOL CDS (~222 nt) are available in the GenBank database, whilst sequence information for CMV is lacking) [1,12,13,19,36,39]. In this study, applying a bigenic PCR approach, we reported significant lengths of DPOL and gB sequences of CMV and LCV from the representative AGM samples (CMV: sample KNA-SD6, and LCV: samples KNA-E4, -N6 and -R15), and could connect the partial DPOL- and gB-CDS of CMV strain KNA-SD6 and LCV strains KNA-E4 and E4-like KNA-N6. Mixed infections with more than one LCV were identified in 2 of the representative AGM samples (KNA-N6 and -R15). To our knowledge, the present study is the first to report analysis of nt/deduced aa sequences from CMV in AGMs and LCV gB CDS from Chlorocebus NHPs.

AGMs have always been the sole NHP species inhabiting the island of St. Kitts. Although the St. Kitts AGMs come in contact with humans, there is no evidence for direct transmission of CMV/LCV between humans and NHPs [2,3,11,16,17,18,19,20,21,22,23,24,25,26]. Therefore, it is likely that CMV and LCV were introduced in St. Kitts during the importation of AGMs from West Africa in the 1600s [27,28], and since then, have evolved in the isolated NHP population, which made it interesting to study the genetic diversity of these viruses. Cospeciations (co-divergence of the virus and host) have been proposed to play important roles during the evolution of CMVs and LCVs, although in this regard, phylogenetically, the branching patterns of OWM LCVs are not always well defined, indicating additional/complex evolutionary events [2,3,11,16,17,18,19,20,21,22,23,24,25,26]. In the present study, the nt and deduced aa sequences (DPOL and gB) from CMV strain KNA-SD6 were most closely related to cognate sequences of CMV (CeBHV5 isolate/strain/s) from grivet monkeys (Figure 3A–C). Although the herpesvirus gB is relatively less conserved than DPOL [2,56], significant differences were observed between the KNA-SD6 DPOL and gB deduced aa identities (~98% and ~87%, respectively) with respective sequences of CeBHV5 isolates/strains. On the other hand, the AGM E4-like LCV nt and concatenated deduced aa sequences were more closely related to cognate sequences of erythrocebus patas LCV1 than those of LCVs from other NHPs (Figure 6A,B). Among the OWMs, AGMs (*Chlorocebus sabeous*) and grivet monkeys (*Chlorocebus aethiops*) are closely related species within the genus *Chlorocebus*, whilst phylogenetically, the genera *Chlorocebus* and *Erythrocebus* cluster together within the tribe *Cercopithecini* (subfamily *Cercopithecinae*, family *Cercopithecidae*) [57]. Taken together, these observations corroborated the concept of cospeciation in the evolution of CMV and LCV.

On the other hand, the partial DPOL of KNA-R15 shared maximum deduced aa identities with MarcGHV1 from a stump-tailed macaque, and phylogenetically, formed a distinct branch within the OWM LCV clade (Figure 5). Based on the hypothetical time tree (constructed using partial DPOL sequences), KNA-R15 appeared to diverge early in evolutionary time from the ancestor of KNA-E4, -N6 and several other LCVs infecting Old World primates (Supplementary Figure S1). Based on deduced aa identities and phylogenetic analysis of partial gB sequences, N6-gB-2 and R15-gB-2 (the additional LCV gB sequences from samples KNA-N6 and -R15, respectively) appeared to be distinct from KNA-E4 and LCVs reported in other OWMs (Figure 7). Phylogenetically, N6-gB-2 clustered separately near an LCV from a hamadryas baboon (Figure 7). Of interest, R15-gB-2 shared a maximum, yet low deduced aa identity of 73.60% with that of PoGHV2 from an orangutan, and phylogenetically, branched outside the OWM LCV clade (Figure 7), indicating that R15-gB-2 might represent a novel LCV, although analysis of the DPOL would be required to validate this observation. These findings pointed towards evolutionary patterns of LCVs that were not synchronous with those of host species, contradicting the virus–host cospeciation hypothesis with regard to evolution of herpesviruses, and indicating complex evolutionary events. Topological incongruences between host and herpesviral phylogenies have been associated with host switches, intrahost speciations and losses, especially losses were shown to be common in beta- and gammaherpesviruses [16]. Therefore, these observations warrant further studies on genetic diversity and evolution of LCVs in AGMs and other NHP species.

Although the present study provided important and first-time insights into the molecular prevalence and genetic diversity/evolution of CMV and LCV in free-roaming AGMs and in captive AGMs with severe respiratory disease, there were limitations: **(i)** this study was based on a small sample size (87 free-roaming AGMs and archival samples from four captive AGMs) and lacked consistency, **(ii)** since sufficient volumes of samples were not available, we could not isolate the AGM herpesviruses and/or subject the CMV/LCV PCR-positive samples to Next Generation Sequencing, which would have been useful to obtain more information on mixed infections and possibly determine the whole genome sequences, or complete DPOL/gB sequences of CMV/LCV strains in the AGM samples, **(iii)** long-length CMV/LCV sequences including those from the St. Kitts AGMs were included in phylogenetic analysis. However, since variable lengths of NHP CMV/LCV sequences were available in the GenBank database, to rule out biases during phylogenetic analysis, we had to trim the AGM CMV/LCV sequences and other published herpesvirus sequences following multiple alignment, and **(iv)** since several of the NHP CMV/LCV were sequenced for a short region of the DPOL/gB, we could not include these sequences in our analysis.

## 4. Materials and Methods

### 4.1. Sampling

The procedures for trapping and obtaining samples from AGMs were approved by the Institutional Animal Care and Use Committee (IACUC) of the Ross University School of Veterinary Medicine (RUSVM), St. Kitts (IACUC #22-5-11), and followed the guidelines mentioned in the RUSVM IACUC approved document “Monkey trapping policies and procedures” (SOP # NHP001, April 2022). During Sep 2022-Feb 2023, nasal (n = 87) and/or rectal (n = 61) samples were obtained from 87 apparently healthy AGMs trapped at different locations on St. Kitts (Figure 1). Some of these free-roaming NHPs were sampled at the trapping site, whilst others were sampled immediately after transportation to the quarantine facility at the Behavioral Science Foundation (BSF), a fully accredited biomedical simian research facility in St. Kitts (Figure 1). The present study also included archival samples (4 nasal swabs and a single oral discharge, collected on 21 and 22 September 2020 and 19 October 2020) from 4 captive AGMs (housed in individual cages at the BSF facility) with clinical signs of severe respiratory disease. The detailed procedures for trapping and sampling AGMs have been described in our recent study on simian AdVs from St. Kitts [40]. Among other pathogens, the AGMs were screened for AdVs using a DNA-dependent DNA polymerase (pol)-, or hexon-based screening PCR assays as described previously [40].

### 4.2. Amplification of Herpesvirus DNA

Total DNA was extracted from the AGM samples using the QIAamp DNA Mini Kit (Qiagen Sciences, Germantown, MD, USA) following the manufacturer’s instructions. The samples were screened for the presence of herpesviruses by a broad-spectrum nested-PCR assay (targeting a short stretch (~215–315 bp) of the herpesvirus DPOL gene) that has been successfully used to detect alpha-, beta- and/or gammaherpesviruses in NHPs and various other host species, including identification of novel herpesviruses [20,49,58,59]. Primers employed in PCRs/Semi-nested PCRs/Nested PCRs to determine the partial DPOL and gB CDS of the AGM herpesviruses were designed in the present study and have been shown in Supplementary Table S1. The PCR reactions were performed using the GoTaq^®^ Green Master Mix (Promega, Madison, WI, USA), or the Platinum™ Taq DNA Polymerase (Invitrogen™, Thermo Fisher Scientific Corporation, Waltham, MA, USA) according to the respective manufacturer’s instructions. To rule out contamination, sterile water was included as the negative control in all PCR reactions.

### 4.3. Nucleotide Sequencing

The PCR products were purified with the Wizard^®^ SV Gel and PCR Clean-Up kit (Promega, Madison, WI, USA) following the protocol described by the manufacturer. Nucleotide sequences were determined by the Sanger dideoxy method using the ABI Prism Big Dye Terminator Cycle Sequencing Ready Reaction Kit (Applied Biosystems, Foster City, CA, USA) on an ABI 3730XL Genetic Analyzer (Applied Biosystems, Foster City, CA, USA). Nucleotide sequences were obtained in both directions.

### 4.4. Analyses of AGM Herpesvirus Sequences

The AGM herpesvirus putative DPOL and gB CDS and corresponding deduced aa sequences were determined using the open reading frame (ORF) finder (https://www.ncbi.nlm.nih.gov/orffinder/, accessed on 15 December 2023), and validated by BLASTN and BLASTP analysis, respectively. Homology search for related herpesvirus sequences was carried out using the standard BLASTN and BLASTP program (accessed on 18 December 2023). Pairwise nt and deduced aa sequence identities (%) were determined using the BLASTN and BLASTP program, respectively, by employing the ‘align two or more sequences’ option (accessed 22 December 2023). For phylogenetic analysis, multiple alignments of nt/deduced aa sequences were performed using the MUSCLE algorithm embedded in the MEGA11 software version 11.0.13 [60], followed by construction of maximum likelihood (ML) trees (using the JTT + G model for deduced aa sequences and the HKY + G model for nt sequences) with 1000 bootstrap replicates, as described previously [1,11,18,22,23,52]. The clustering patterns of the AGM herpesvirus sequences in ML trees were confirmed with other mathematical models of substitutions (LG + G and Poisson models for aa sequences and Kimura 2-parameter and GTR models for nt sequences) as well as by the neighbor-joining (NJ) method. The LCV nt sequences (partial DPOL CDS-intergenic region-partial gB CDS) from AGMs were subjected to recombination analysis using the RDP4 program with default parameters [61]. A herpesvirus sequence was considered as a recombinant if it was validated by two, or more detection methods (3Seq, BOOTSCAN, CHIMERA, GENECONV, MAXCHI, RDP and SISCAN) with a highest acceptable *p*-value of *p* < 0.01 with Bonferroni’s correction [40,61].

### 4.5. GenBank Accession Numbers

The GenBank accession numbers for the AGM herpesvirus sequences determined in this study are PP098626-PP098652 and PP238509.

## Figures and Tables

**Table 1:** Details of the African green monkeys (AGMs) that tested positive for herpesviruses in the Caribbean Island of St. Kitts (KNA).

AGM/Sample Number	Age, Gender	Herpesvirus-Positive Sample	Date of Sample Collection	Sampling/Trapping Location in St. Kitts	*Genus* ^1^
KNA-E4 ^2^	Juvenile, female	Nasal swab	19 Oct 2020	Behavioral Science Foundation ^3^	*Lymphocryptovirus*
KNA-E5 ^4^	Not available	Nasal swab	22 Sep 2020	Behavioral Science Foundation ^3^	*Cytomegalovirus*
KNA-E5-O ^4^	Not available	Oral discharge	22 Sep 2020	Behavioral Science Foundation ^3^	*Lymphocryptovirus*
KNA-E6 ^2^	Juvenile, female	Nasal swab	21 Sep 2020	Behavioral Science Foundation ^3^	*Lymphocryptovirus*
KNA-R2	Juvenile, male	Rectal swab	24 Sep 2022	Monkey hill ^5,6^	Sequence lacked quality ^7^
KNA-R3	Adult, female	Rectal swab	24 Sep 2022	Monkey hill ^5,6^	Sequence lacked quality ^7^
KNA-N5	Juvenile, male	Nasal swab	24 Sep 2022	Monkey hill ^5,6^	*Lymphocryptovirus*
KNA-N6	Adult, female	Nasal swab	24 Sep 2022	Monkey hill ^5,6^	*Lymphocryptovirus*
KNA-N10	Adult, female	Nasal swab	15 Dec 2022	Monkey hill ^5,6^	*Cytomegalovirus*
KNA-N11	Adult, female	Nasal swab	15 Dec 2022	Monkey hill ^5,6^	*Lymphocryptovirus*
KNA-R12	Juvenile, female	Rectal swab	15 Dec 2022	Monkey hill ^5,6^	*Lymphocryptovirus*
KNA-R15	Juvenile, male	Rectal swab	15 Dec 2022	Monkey hill ^5,6^	*Lymphocryptovirus*
KNA-R18	Adult, male	Rectal swab	19 Dec 2022	Cedar grove ^6,8^	*Lymphocryptovirus*
KNA-N21	Adult, male	Nasal swab	19 Dec 2022	Cedar grove ^6,8^	*Cytomegalovirus*
KNA-N32	Adult, male	Nasal swab	29 Dec 2022	Green hill ^6,8^	Sequence lacked quality ^7^
KNA-N33	Adult, male	Nasal swab	29 Dec 2022	Green hill ^6,8^	*Lymphocryptovirus*
KNA-N36	Adult, male	Nasal swab	29 Dec 2022	Green hill ^6,8^	*Lymphocryptovirus*
KNA-N37	Adult, male	Nasal swab	29 Dec 2022	Green hill ^6,8^	*Lymphocryptovirus*
KNA-N44 ^9^	Adult, male	Nasal swab	10 Jan 2023	Fountain mountain ^8,10^	Sequence lacked quality ^7^
KNA-R44 ^9^	Adult, male	Rectal swab	10 Jan 2023	Fountain mountain ^8,10^	Sequence lacked quality ^7^
KNA-R49	Adult, female	Rectal swab	10 Jan 2023	Fountain mountain ^8,10^	Sequence lacked quality ^7^
KNA-18-2R	Adult, female	Rectal swab	18 Jan 2023	Harris mountain forest ^8,10^	Sequence lacked quality ^7^
KNA-1810	Adult, male	Nasal swab	18 Jan 2023	Harris mountain forest ^8,10^	*Lymphocryptovirus*
KNA-DM9	Juvenile, female	Nasal swab	24 Jan 2023	Dale mountain ^6,8^	*Lymphocryptovirus*
KNA-SD6	Adult, female	Nasal swab	1 Feb 2023	Saddlers ^6,8^	*Cytomegalovirus*
KNA-SD13	Adult, female	Nasal swab	1 Feb 2023	Saddlers ^6,8^	*Lymphocryptovirus*
KNA-SD14	Adult, female	Nasal swab	1 Feb 2023	Saddlers ^6,8^	*Lymphocryptovirus*
KNA-SD15	Adult, female	Nasal swab	1 Feb 2023	Saddlers ^6,8^	Sequence lacked quality ^7^

**^1^** Based on BLASTN analysis of the partial DNA polymerase (DPOL) coding sequence obtained from the screening PCR (pan-herpesvirus DPOL nested PCR) assay. **^2^** The AGMs died after exhibiting clinical signs of acute respiratory disease. **^3^** The samples were obtained from captive AGMs (kept in individual cages) at the Behavioral Science Foundation (BSF), Mills estate, St. Kitts. **^4^** The oral and nasal samples were obtained from the same AGM. **^5^** The AGM was sampled at the trapping site. **^6^** The AGM was trapped in/near human habitat. **^7^** Although BLASTN analysis of the partial DPOL coding sequence revealed homology with herpesviruses, the nucleotide sequence lacked quality (Phred value < 40) and was not used for further analysis. **^8^** The AGM was sampled immediately after transportation from the trapping site **^8^** to the BSF quarantine facility. **^9^** The nasal and rectal samples were obtained from the same AGM. **^10^** The AGM was trapped in the wild.

## Data Availability

The data presented in this study are available in this article, Supplementary Table S1 and Supplementary Figure S1.

## Supplementary Materials

The supporting information can be downloaded at: https://www.mdpi.com/article/10.3390/ijms25063272/s1. References [16,60,62,63] are cited in the Supplementary Materials.
