# Supplementary material for: Molecular Detection and Genetic Diversity of Cytomegaloviruses and Lymphocryptoviruses in Free-Roaming and Captive African Green Monkeys (Chlorocebus sabaeus)
Source: Int J Mol Sci. 2024 Mar 14;25(6):3272. doi: 10.3390/ijms25063272 (PMC10970604; doi:10.3390/ijms25063272)
Supplement: Supplementary file 1 [file ijms-25-03272-s001.zip › Supplementary figure S1.pptx]

## Slide 1
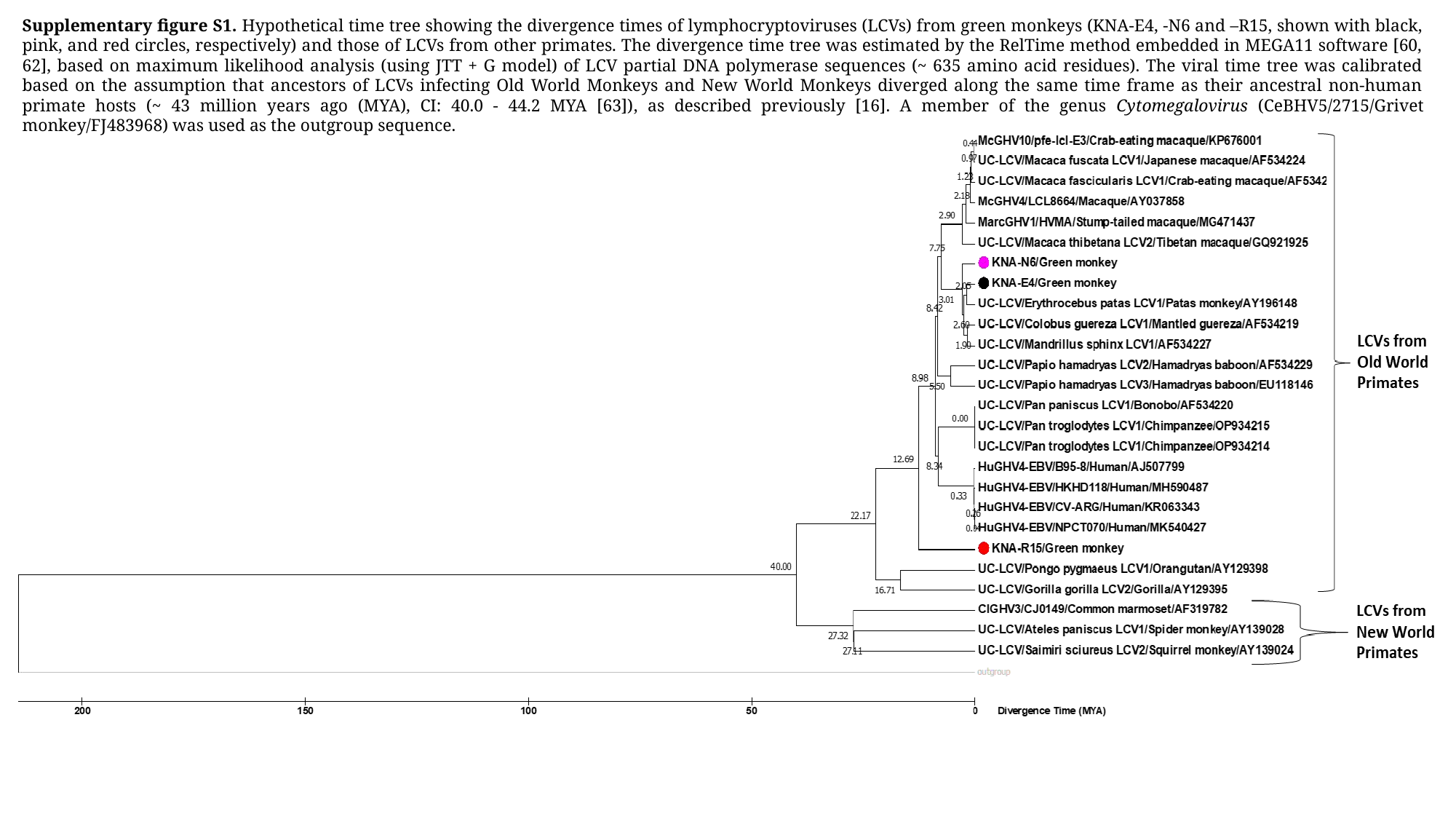

Supplementary figure S1. Hypothetical time tree showing the divergence times of lymphocryptoviruses (LCVs) from green monkeys (KNA-E4, -N6 and –R15, shown with black, pink, and red circles, respectively) and those of LCVs from other primates. The divergence time tree was estimated by the RelTime method embedded in MEGA11 software [60, 62], based on maximum likelihood analysis (using JTT + G model) of LCV partial DNA polymerase sequences (~ 635 amino acid residues). The viral time tree was calibrated based on the assumption that ancestors of LCVs infecting Old World Monkeys and New World Monkeys diverged along the same time frame as their ancestral non-human primate hosts (~ 43 million years ago (MYA), CI: 40.0 - 44.2 MYA [63]), as described previously [16]. A member of the genus Cytomegalovirus (CeBHV5/2715/Grivet monkey/FJ483968) was used as the outgroup sequence.
