# Supplementary material for: Molecular Detection and Genetic Diversity of Cytomegaloviruses and Lymphocryptoviruses in Free-Roaming and Captive African Green Monkeys (Chlorocebus sabaeus)
Source: Int J Mol Sci. 2024 Mar 14;25(6):3272. doi: 10.3390/ijms25063272 (PMC10970604; doi:10.3390/ijms25063272)
Supplement: Supplementary file 1 [file ijms-25-03272-s001.zip › Supplementary table S1.docx]

| ***Betaherpesvirus/Cytomegalovirus***  **Supplementary Table S1.** Primers employed in PCR assays to amplify the viral genomes of betahepesviruses and gammaherpesviruses detected in green monkeys (GM) on the Island of St. Kitts. DPOL, *herpesvirus DNA polymerase*; gB, *herpesvirus envelope glycoprotein B*; nt, *nucleotide*; ORF, *open reading frame*. | | | | | | |
| --- | --- | --- | --- | --- | --- | --- |
| **GM Sample** | **Target** | **PCR name** | **Primer name** | **Primer sequence (5’-3’)** | **Primer position** | **Comments** |
| **KNA-SD6** | DPOL ORF | DPOL-1  (Nested PCR) | BPA1F | TCCAACCGCGTCAAAATGTACTTG | nt 75900-nt 75923 | Primer nt positions are those of Cercopithecine betaherpesvirus 5 (GenBank accession number FJ483969). The primers were designed following alignment of the complete genomic sequences of Cercopithecine betaherpesvirus 5 strains Colburn and 2715. |
|  |  |  | BPA1R | TTACYTGGCTGGTGCCCGAAAACC | nt 77075-nt 77052 |  |
|  |  |  | BPA2F | GCGTTGATGTTGTATCCAGTCACG | nt 75933-nt 75956 |  |
|  |  |  | BPA2R | CGCAGAAGACGTTTCTGGAGC | nt 77025-nt 77005 |  |
|  |  | DPOL-2  (Nested PCR) | BPB1F | GTCGACGTGCGCGTGAGCATG | nt 74916-nt 74936 |  |
|  |  |  | BPB1R | GTTACGAAACTGGGGGAACGGG | nt 76134-nt 76113 |  |
|  |  |  | BPB2F | ACCGATACGGGTGATGCTGGC | nt 74942-nt 74962 |  |
|  |  |  | BPB2R | GCACGGTGTTTGGAACATCGG | nt 76101-nt 76081 |  |
|  | gB ORF | gB-1 | BGF1 | TGCAAAGCCAAMAGCATCT | nt 77250-nt 77268 | Primer nt positions are those of Cercopithecine betaherpesvirus 5 (FJ483969). The primers were designed following multiple alignment of the complete genomic sequences of Cercopithecine betaherpesvirus 5 strains Colburn and 2715 with other non-human primate cytomegaloviruses. |
|  |  |  | BGR1 | TATGTKGACTATSTGTACAAAAGA | nt 77881-nt 77858 |  |
|  |  | gB-2 | BGF2 | TCSGTGTTTTCCARYGGATCAAT | nt 77784-nt 77806 |  |
|  |  |  | BGR2 | TTTTGGGAACAATCRGAACG | nt 78727-nt 78708 |  |
|  | DPOL ORF-intergenic region-gB ORF | Intergenic  (Nested PCR) | SD6-link-F1 | TGACGGAGGCAAAGGACACG | nt 1851-nt 1870 | Primer nt positions are those of strain SD6. The primer sequences were designed from the partial DPOL and gB sequences obtained from strain SD6. |
|  |  |  | SD6-link-R1 | CGCGCCTGTAAAAGAAACTCC | nt 2367-nt 2347 |  |
|  |  |  | SD6-link-F2 | CACGCCATGTCATTATCAAGCAC | nt 1873-nt 1895 |  |
|  |  |  | SD6-link-R2 | ATGAAGAAAGCCTCTATTCCAGC | nt 2332-nt 2310 |  |
| ***Gammaherpesvirus/Lymphocryptovirus*** | | | | | | |
| **GM Sample** | **Target** | **PCR name** | **Primer name** | **Primer sequence (5’-3’)** | **Primer position** | **Comments** |
| **KNA-E4** | DPOL ORF | DPOL-A  (Nested PCR) | GPA1F | TCAAAGTTGGCCACGTTGTAGC | nt 151180-nt 151201 | **^1^** Primer nt positions are those of Macacine gammaherpesvirus 4 (GenBank accession number AY037858). |
|  |  |  | GPA1R | CCCTTTCCTAAGACCTAATAAAGGCC | nt 152307-nt 152282 |  |
|  |  |  | GPA2F | GATGAGCTGGAAGAAGGCGTAG | nt 151230-nt 151251 |  |
|  |  |  | GPA2R | CAAGGAGTACCTGCGTCTCATTCC | nt 152265-nt 152242 |  |
|  |  | DPOL-B  (Nested PCR) | GPB1F | GTCTCGGCGATGGAGAGGCAGGG | nt 150214-nt 150236 |  |
|  |  |  | GPB1R | GAGTGTCTGGGGGAGGAGGGCT | nt 151439-nt 151418 |  |
|  |  |  | GPB2F | GTTGCACGTGCACTTGATGGCCAG | nt 150276-nt 150299 |  |
|  |  |  | GPB2R | CACGGCCACCAACGAGGCTGAC | nt 151413-nt 151392 |  |
|  | DPOL ORF-intergenic region-gB ORF | Intergenic  (Nested PCR) | Link-1F | TTCTGGCCTAGCTGCCACCTC | nt 1750-nt 1770 | Primer nt positions are those of strain E4. The primer sequences were designed from the partial DPOL and gB sequences obtained from strain E4. |
|  |  |  | Link-1R | GGACAATGCGGTCTCAAACAACAG | nt 2544-nt 2521 |  |
|  |  |  | Link-2F | CCACCACATGCCCTTTCCATC | nt 1786-nt 1806 |  |
|  |  |  | Link-2R | GGCCTAGGGGAACTCATGGAC | nt 2504-nt 2484 |  |
|  | gB ORF | gB-A  (Nested PCR) | GGA1F | CGCAGGGAGTCRTAGGCAAA | nt 153507-nt 153526 | **^1^** Primer nt positions are those of Macacine gammaherpesvirus 4 (AY037858). |
|  |  |  | GGA1R | GTCTGCGAGCTCTCCAGCCACG | nt 154780-nt 154759 |  |
|  |  |  | GGA2F | TGGCVGGATTGTTGAGGGTGCC | nt 153541-nt 153562 |  |
|  |  |  | GGA2R | GACCTSTTCCGCTTCTCCTCGG | nt 154756-nt 154735 |  |
|  |  | gB-B  (Nested PCR) | GGB1F | CTCATGYAGCGCTAACATGATGG | nt 152498-nt 152520 |  |
|  |  |  | GGB1R | GGAGGATTGTTATTYGCTTGGCT | nt 153802-nt 153780 |  |
|  |  |  | GGB2F | TAGAGCATCTGCACCGGCTGC | nt 152610-nt 152630 |  |
|  |  |  | GGB2R | GCTTGGCTRCCTCTGACCCC | nt 153787-nt 153768 |  |
|  |  |  |  |  |  |  |
| **KNA-N6** | DPOL ORF | DPOL-A  (Nested PCR) | GPA1F | TCAAAGTTGGCCACGTTGTAGC | nt 151180-nt 151201 | **^1^** Primer nt positions are those of Macacine gammaherpesvirus 4 (AY037858). |
|  |  |  | GPA1R | CCCTTTCCTAAGACCTAATAAAGGCC | nt 152307-nt 152282 |  |
|  |  |  | GPA2F | GATGAGCTGGAAGAAGGCGTAG | nt 151230-nt 151251 |  |
|  |  |  | GPA2R | CAAGGAGTACCTGCGTCTCATTCC | nt 152265-nt 152242 |  |
|  |  | DPOL-B  (Nested PCR) | GPB1F | GTCTCGGCGATGGAGAGGCAGGG | nt 150214-nt 150236 |  |
|  |  |  | GPB1R | GAGTGTCTGGGGGAGGAGGGCT | nt 151439-nt 151418 |  |
|  |  |  | GPB2F | GTTGCACGTGCACTTGATGGCCAG | nt 150276-nt 150299 |  |
|  |  |  | GPB2R | CACGGCCACCAACGAGGCTGAC | nt 151413-nt 151392 |  |
|  | DPOL ORF-intergenic region-gB ORF  (E4-like N6 gB ORF) | Intergenic  (Nested PCR) | Link-1F | TTCTGGCCTAGCTGCCACCTC | nt 1750-nt 1770 | Primer nt positions are those of strain E4. The primer sequences were designed from the partial DPOL and gB sequences obtained from strain E4. |
|  |  |  | Link-1R | GGACAATGCGGTCTCAAACAACAG | nt 2544-nt 2521 |  |
|  |  |  | Link-2F | CCACCACATGCCCTTTCCATC | nt 1786-nt 1806 |  |
|  |  |  | Link-2R | GGCCTAGGGGAACTCATGGAC | nt 2504-nt 2484 |  |
|  | E4-like N6 gB ORF | gB-A  (Nested PCR) | GGA1F | CGCAGGGAGTCRTAGGCAAA | nt 153507-nt 153526 | **^1^** Primer nt positions are those of Macacine gammaherpesvirus 4 (AY037858). PCR amplicon yielded a E4-like gB sequence. |
|  |  |  | GGA1R | GTCTGCGAGCTCTCCAGCCACG | nt 154780-nt 154759 |  |
|  |  |  | GGA2F | TGGCVGGATTGTTGAGGGTGCC | nt 153541-nt 153562 |  |
|  |  |  | GGA2R | GACCTSTTCCGCTTCTCCTCGG | nt 154756-nt 154735 |  |
|  |  | N6/E4-like gB  (Semi-nested PCR) | E4-3172F | CGCAAGGAGTCGTACGCGAAC | nt 3172-nt 3192 | Primer nt positions are those of strain E4. The primer sequences were designed from the gB sequence obtained from strain E4 and used in semi-nested PCR assay to obtain a longer E4-like N6 gB sequence. |
|  |  |  | E4-3200F | GGATGGTGGCCGGATTGTTG | nt 3200-nt 3219 |  |
|  |  |  | E4-4176R | CTACCAGTGTTACAACTCTGTTC | nt 4176-nt 4154 |  |
|  | N6-gB-2 ORF | gB-B  (Nested PCR) | GGB1F | CTCATGYAGCGCTAACATGATGG | nt 152498-nt 152520 | **^1^** Primer nt positions are those of Macacine gammaherpesvirus 4 (AY037858). The sequence obtained from the gB-B PCR did not overlap with the gB-A sequence (E4-like N6 gB sequence). |
|  |  |  | GGB1R | GGAGGATTGTTATTYGCTTGGCT | nt 153802-nt 153780 |  |
|  |  |  | GGB2F | TAGAGCATCTGCACCGGCTGC | nt 152610-nt 152630 |  |
|  |  |  | GGB2R | GCTTGGCTRCCTCTGACCCC | nt 153787-nt 153768 |  |
|  |  |  |  |  |  |  |
| **KNA-R15** | DPOL ORF | DPOL-A  (Nested PCR) | GPA1F | TCAAAGTTGGCCACGTTGTAGC | nt 151180-nt 151201 | **^1^** Primer nt positions are those of Macacine gammaherpesvirus 4 (AY037858). |
|  |  |  | GPA1R | CCCTTTCCTAAGACCTAATAAAGGCC | nt 152307-nt 152282 |  |
|  |  |  | GPA2F | GATGAGCTGGAAGAAGGCGTAG | nt 151230-nt 151251 |  |
|  |  |  | GPA2R | CAAGGAGTACCTGCGTCTCATTCC | nt 152265-nt 152242 |  |
|  |  | DPOL-B  (Nested PCR) | GPB1F | GTCTCGGCGATGGAGAGGCAGGG | nt 150214-nt 150236 |  |
|  |  |  | GPB1R | GAGTGTCTGGGGGAGGAGGGCT | nt 151439-nt 151418 |  |
|  |  |  | GPB2F | GTTGCACGTGCACTTGATGGCCAG | nt 150276-nt 150299 |  |
|  |  |  | GPB2R | CACGGCCACCAACGAGGCTGAC | nt 151413-nt 151392 |  |
|  | E4-like R15 gB ORF | gB-A  (Nested PCR) | GGA1F | CGCAGGGAGTCRTAGGCAAA | nt 153507-nt 153526 | **^1^** Primer nt positions are those of Macacine gammaherpesvirus 4 (AY037858). PCR amplicon yielded a E4-like gB sequence. |
|  |  |  | GGA1R | GTCTGCGAGCTCTCCAGCCACG | nt 154780-nt 154759 |  |
|  |  |  | GGA2F | TGGCVGGATTGTTGAGGGTGCC | nt 153541-nt 153562 |  |
|  |  |  | GGA2R | GACCTSTTCCGCTTCTCCTCGG | nt 154756-nt 154735 |  |
|  |  | R15/E4-like gB | E4-3200F | GGATGGTGGCCGGATTGTTG | nt 3200-nt 3219 | Primer nt positions are those of strain E4. The primer sequences were designed from the gB sequence obtained from strain E4 and used in PCR assay to obtain a longer E4-like R15 gB sequence. |
|  |  |  | E4-4222R | AAACGTACTCGGTGGAGAGCC | nt 4222-nt 4202 |  |
|  | R15-gB-2 ORF | gB-B  (Nested PCR) | GPB1F | GTCTCGGCGATGGAGAGGCAGGG | nt 150214-nt 150236 | **^1^** Primer nt positions are those of Macacine gammaherpesvirus 4 (AY037858). The sequence obtained from the gB-B PCR did not overlap with the gB-A sequence (E4-like R15 gB sequence). |
|  |  |  | GPB1R | GAGTGTCTGGGGGAGGAGGGCT | nt 151439-nt 151418 |  |
|  |  |  | GPB2F | GTTGCACGTGCACTTGATGGCCAG | nt 150276-nt 150299 |  |
|  |  |  | GPB2R | CACGGCCACCAACGAGGCTGAC | nt 151413-nt 151392 |  |

**^1^** The primers were designed following multiple alignment of the complete genomic sequences of reference lymphocryptovirus strains from human and non-human primates.
